# Comparison of the benefits of celecoxib combined with anticancer therapy in advanced non-small cell lung cancer: A meta-analysis

**DOI:** 10.7150/jca.35003

**Published:** 2020-01-20

**Authors:** Wei Zhang, Lilan Yi, Jie Shen, Hongman Zhang, Peng Luo, Jian Zhang

**Affiliations:** Department of Oncology, Zhujiang Hospital, Southern Medical University, 253 Industrial Avenue, Guangzhou, 510282, Guangdong, People's Republic of China.

**Keywords:** celecoxib, non-small cell lung cancer, comprehensive therapy, meta-analysis

## Abstract

**Background:** Studies have reported that advanced NSCLC benefits from celecoxib combined with systematic treatment. However, the optimal combination with different treatments remains unclear. A meta-analysis was conducted to explore treatment combinations.

**Methods**: We searched the relevant literature via PubMed, EMBASE, the Cochrane Library and PMC. The data for the overall response rate (ORR), overall survival (OS), progression-free survival (PFS), and adverse effects were obtained. Subgroup analysis was performed according to the treatment pattern. Statistical analyses were carried out using Review Manager 5.3 software.

**Results**: A total of 18 eligible studies were included, with 1178 advanced NSCLC patients. Subgroup analysis revealed that celecoxib combined with chemotherapy or tyrosine kinase inhibitors (TKIs) significantly increased the ORR, with no significant difference between the two groups. Celecoxib combined with chemotherapy improved OS-6 (OR=0.65, 95% CI 0.59-0.71, P<0.001), while OS-6 was not changed with celecoxib combined with TKIs (OR=0.53, 95% CI 0.31-0.73, P=0.82). Differences were apparent between the chemotherapy and TKIs regarding OS-6 (P=0.0392). Celecoxib combined with chemotherapy significantly prolonged OS-12 (OR=0.39, 95% CI 0.33-0.45, P<0.001). In terms of OS-12, there was no significant improvement when celecoxib was combined with radiotherapy or TKIs. Celecoxib combined with chemotherapy or TKIs significantly improved PFS-6 and PFS-12, with no obvious difference in terms of PFS between the two groups. Additionally, celecoxib combined with chemotherapy or TKI treatment increased the incidence of adverse events, with no significant differences between the two groups.

**Conclusions**: Celecoxib combined with chemotherapy or TKIs improved the ORR, with no significant differences between the two groups. In terms of OS, celecoxib combined with chemotherapy was superior to TKIs or radiotherapy. Accordingly, celecoxib combined with chemotherapy increased hematological toxicity and cardiovascular events.

## Introduction

Lung cancer is ranked as the number one cause of cancer-related mortality worldwide, with 1.6 million deaths per year. Non-small cell lung cancer (NSCLC) makes up >75% of all lung cancer cases, with a 5-year survival rate of less than 20%; therefore, NSCLC is the focus of most lung cancer studies.^1^,^2^ The National Comprehensive Cancer Network (NCCN) recommends platinum-based chemotherapy as the first-line agent for patients with advanced NSCLC with non-driver genes.^3^ However, chemotherapy for patients with advanced NSCLC is unsatisfactory due to potential complications and suboptimal survival rates. The median survival time for patients treated with carboplatin plus paclitaxel is unsatisfactory, and this regimen causes grade 3 or higher toxicities, including nausea and neurological diseases.^4^ Consequently, the NCCN guideline recommends erlotinib as the primary therapy for patients with an EGFR mutation or chemotherapeutic failure.^5^-^7^ Additionally, crizotinib is recommended as a first-line treatment for patients with ALK and ROS1 mutations.^8^-^10^ Preclinical studies have also demonstrated that small-molecule EGFR tyrosine kinase inhibitors (TKIs) are superior to cytotoxic chemotherapy in the treatment of patients with EGFR-mutant NSCLC, further increasing the overall response rate (ORR).^11^ In similar studies, gefitinib and erlotinib improved progression-free survival (PFS) (HR=0.43, 95% CI 0.38-49, P<0.001) compared with platinum-based chemotherapy.^12^ Although small-molecule inhibitors have shown excellent clinical efficacy in patients with gene mutations, disease progression is inevitable because of the emergence of acquired resistance. Brain radiotherapy is regarded as the preferential therapy for patients with multiple brain metastases (BMs) of NSCLC and can alleviate symptoms and prolong survival.^13^ However, external radiation therapy significantly increases adverse events (AEs), including radiation pneumonitis.^14^,^15^ Despite great advances in treatments with anticancer therapy, we still need to optimize treatment strategies to improve the clinical efficacy of therapy for advanced NSCLC.

Recently, comprehensive regimens for advanced NSCLC have attracted the attention of scientists. Relative to monotherapy, comprehensive therapy can achieve dual effects, obtain excellent clinical efficacy, significantly improve the local control rate and prolong survival.^16^-^18^ A meta-analysis related to combination therapy has shown that platinum + paclitaxel combined with bevacizumab significantly prolongs PFS (HR=0.57, 95% CI 0.46-0.71, P<0.00001), improves overall survival (OS) (HR=0.81, 95% CI 0.71-0.92, P=0.0009) and achieves a higher ORR (RR = 2.06, 95% CI 1.73-2.44, P< 0.00001) than platinum + paclitaxel alone.^19^ A similar study highlights the better clinical efficacy of TKIs combined with bevacizumab relative to TKIs alone for advanced NSCLC.^20^ An and his colleagues have shown that cisplatin combined with endostar significantly improves the one-year survival rate of patients (RR=1.70, 95% CI 1.07-2.89, P<0.05) compared with chemotherapy alone.^21^ Additionally, Wang et al. have indicated that radiotherapy combined with EGFR-TKI increases the ORR (RR=1.32, 95% CI 1.13-1.55) and improves OS (HR = 0.72, 95% CI 0.59-0.89) compared with monotherapy.^22^ Based on the above observations, an exploration of comprehensive treatments for advanced NSCLC would be meaningful.

With constant investigations of anticancer treatment, evidence has surfaced indicating that the cancer incidence is significantly correlated with inflammation.^23^ Patel et al. have suggested that cyclooxygenase-2 (COX-2) is overexpressed in lung adenocarcinomas, accounting for more than 70% of these cancers.^24^,^25^ Moreover, similar studies have demonstrated that selective COX-2 inhibitors can inhibit the growth of lung cancer cells and boost the efficacy of chemotherapy in advanced NSCLC.^26^ Celecoxib is a new nonsteroidal anti-inflammatory drug (NSAID) and a selective COX-2 inhibitor that can prevent cancerization* in vivo* and *in vitro* and reduce the growth rates of various tumors.^27^ Data by Jiang et al.^28^ confirm that COX-2 expression is a prognostic indicator for the treatment of advanced NSCLC. However, although current evidence indicates that the COX-2 inhibitor is widely considered as an ancillary drug that can be combined with different anticancer treatments for advanced NSCLC, there is no evidence that treatment of advanced NSCLC with celecoxib alone has a better clinical benefit than the combination treatment. Some clinical studies have suggested that celecoxib combined with various anticancer therapies can achieve excellent clinical efficacy in patients with advanced NSCLC. Platinum-based chemotherapy combined with celecoxib has a significantly improved objective response rate (38% vs 30%) compared with chemotherapy alone.^29^ In addition, studies with celecoxib in combination with erlotinib have shown a significant prolongation of PFS in patients with high COX-2 expression (5.6 vs 2.0 months, P=0.048).^30^ Similarly, docetaxel combined with celecoxib can significantly improve PFS (HR=0.43, 95% CI 0.38-0.49, P<0.001).^31^ Our previous study also demonstrates that celecoxib combined with systematic treatment benefits patients with advanced NSCLC.^32^ Although the prognosis of patients treated with celecoxib combined with anticancer treatment for advanced NSCLC is significantly improved, the optimal combination with different treatments has yet to be fully determined. To address this problem, we performed this systematic review and meta-analysis to compare the clinical efficacy of celecoxib in combination with anticancer therapy in patients with advanced NSCLC.

## Materials and methods

Our meta-analysis was performed in accordance with the Preferred Reporting Items for Systematic reviews and Meta-Analyses (PRISMA) protocol.^33^

### Search strategy

We retrieved relevant studies published between Jan 1, 2001, and July 13, 2019 by searching the PubMed, EMBASE, Cochrane Library and PMC databases. We applied the following MeSH Database and limited search terms (title, abstract): carcinoma, non-small cell lung, non-small cell lung cancer, and NSCLC; Celecoxib, cyclooxygenase-2 inhibitor, COX-2 inhibitor, and COX-2 inhibition; and clinical trial. Moreover, the reference list of primary articles published in English was manually searched to obtain more eligible articles. Two authors (WZ and LLY) independently selected the clinical trials for the meta-analysis.

### Literature selection and exclusion

The primary criteria for inclusion were as follows: (a) patients had histologically or cytologically confirmed advanced NSCLC with celecoxib treatment; (b) clinical trials that reported the outcomes of celecoxib in combination with multiple anticancer therapies including chemotherapy, TKIs or radiotherapy for patients with advanced NSCLC; (c) trials focused on comparing the optimal celecoxib combination with different treatments for patients with advanced NSCLC; (d) patients with adequate organ and bone marrow function with an Eastern Tumor Cooperative Group (ECOG) performance status of 0-2; (e) eligible patients were adults (≥18 years) and the number of patients with advanced NSCLC was >20; and (f) the outcomes were efficacy (overall survival, progression-free survival, tumor response) and toxicity (incidence of adverse effects (AEs).

The main exclusion criteria were as follows: (a) the study was a case study, literature review, animal study or prospective retrospective study; (b) unrelated studies with incomplete data; (c) studies from which data could not be extracted or obtained by contacting the author; (d) studies not published in English; and (e) studies with duplicate or previously published data.

### Data extraction

Two investigators (WZ and LLY) independently extracted the relevant data, and disagreements were resolved by the third investigator (PL). The main relevant information, namely, the research design, patient characteristics, interventions and results, was collected from each selected study. The primary endpoints were as follows: ORR, OS-12, OS-6, PFS-6, and PFS-12. For survival data that were not described in detail in the text, we applied the software Engauge Digitizer version 4.1 (http://digitizer.sourceforge.net/) for data extraction. The AEs (grade≥III) included hematological and nonhematological AEs.

### Statistical analysis

Statistical analysis was conducted with RevMan 5.3 software (Cochrane Library, Oxford, UK). For dichotomous variables, we also calculated a single rate with the pooled estimates of the odds ratio (OR) with a random-effects model. Our results were calculated with the ratio class method, and the final results were obtained by conversion.^34^ If heterogeneity between studies per I^2^ statistics was determined to be more than 50%, indicating moderate-to-high heterogeneity,^35^ the random-effects model was used. Otherwise, the fixed-effects model was used. Subgroup analysis was performed in accordance with the celecoxib combination pattern to explore potential heterogeneity sources. For the primary endpoints, we compared the rates of the two groups to analyze the optimal combination with different treatments.^36^ A P-value less than 0.05 was considered statistically significant. In addition, the Begg's funnel plot and Egger's test (Stata 12.0, Stata Corporation, College Station, TX, USA) were used to determine the possibility of publication bias. Moreover, the trim-and-fill method was used to assess the impact of publication bias on the interpretation of the results.^37^ Furthermore, we applied the Kaplan-Meier method with SPSS 20 to establish OS and PFS survival curves correlations with celecoxib in combination with chemotherapy or TKIs. All p-values were two-sided, and a P-value ≤ 0.05 was deemed statistically significant.

## Results

### Studies characteristics

**Figure 1:**
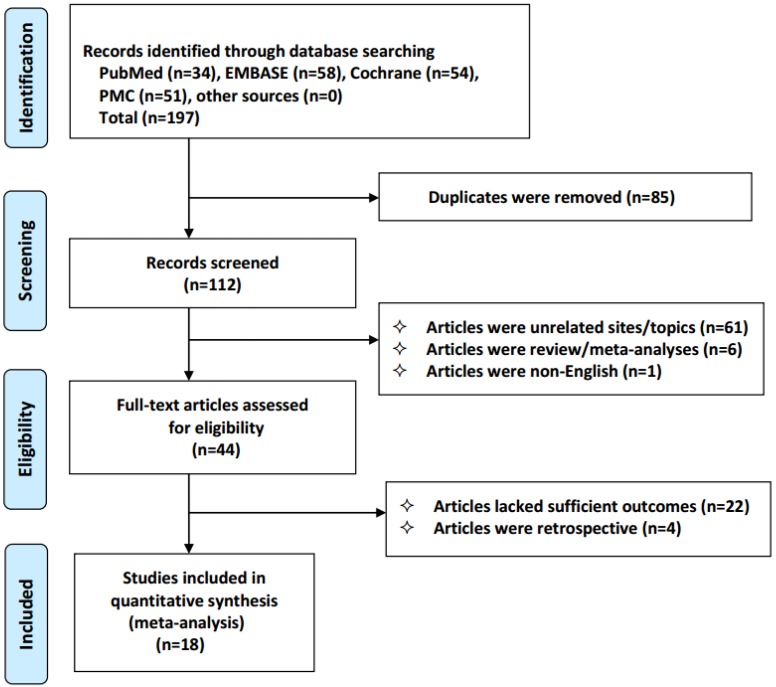
Flowchart of the selection process in accordance with the PRISMA 2009 checklist.

We identified 197 studies, of which 18 qualified articles were included in our analysis. The following studies were disqualified: duplicate studies (n=85), unrelated studies (n=61); reviews and meta-analyses (n=6); non-English studies (n=1); studies lacking clinical outcomes (n=22); and retrospective studies (n=4). The selection process for the results is shown in Figure 1. The analysis included 1178 patients with advanced NSCLC who received celecoxib combined with anticancer therapy. Only one of the included studies examined treatment with celecoxib in combination with radiotherapy, and the others examined treatment with chemotherapy or tyrosine kinase inhibitors. The details of the main features of the study are provided (Table 1).

### Tumor response

**Figure 2:**
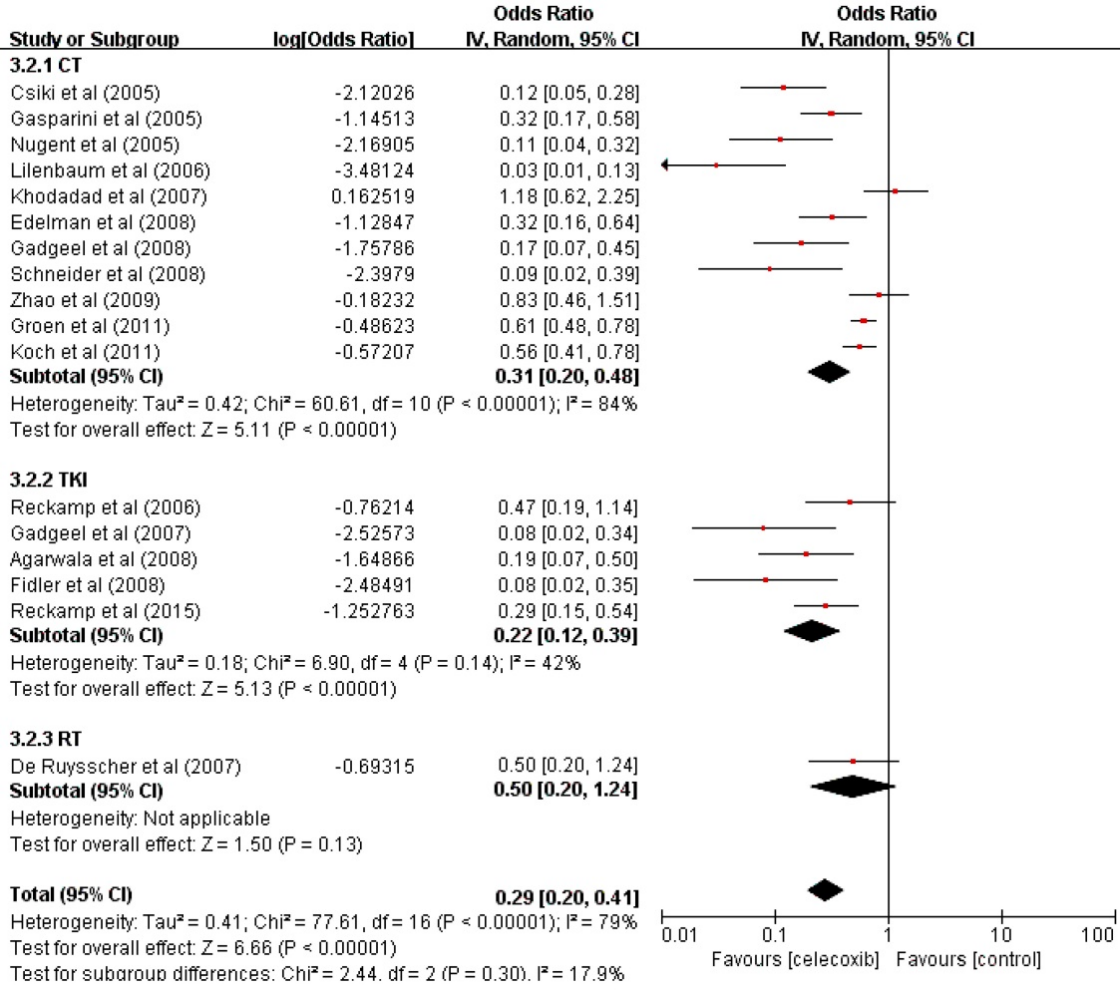
Forest plots of the overall response rate (ORR) for celecoxib treatment combined with systematic therapy. Abbreviations: OR: odds ratio; IV: inverse variance; CI: confidence interval; CT: chemotherapy; TKIs: tyrosine kinase inhibitors; RT: radiotherapy.

The data of seventeen clinical studies that reported the ORR of 283 patients were pooled, and the odds ratio of ORR was estimated (OR=0.22, 95% CI 0.17-0.29, P<0.001) (Table 2) with the random-effect model (I^2^=79%, P<0.001) (Figure 2).

The ORR was significantly increased compared with that found in previous studies. To determine whether the increased ORR was due to the use of celecoxib in combination with other different treatments, we performed a further subgroup analysis of the ORR according to the treatment pattern. These results supported the idea of an increase in the ORR of patients treated with celecoxib and chemotherapy (OR=0.24, 95% CI 0.17-0.32, P<0.001) or TKIs (OR=0.18, 95% CI 0.11-0.28, P<0.001), with no significant difference between the two groups in terms of the ORR (24% vs 18%, P=0.4907). Celecoxib combined with radiotherapy did not improve the ORR (OR=0.33, 95% CI 0.17-0.55, P=0.13). Thus, celecoxib in combination with chemotherapy or TKIs demonstrated improved ORR compared to celecoxib in combination with radiotherapy.

### Survival

**Figure 3:**
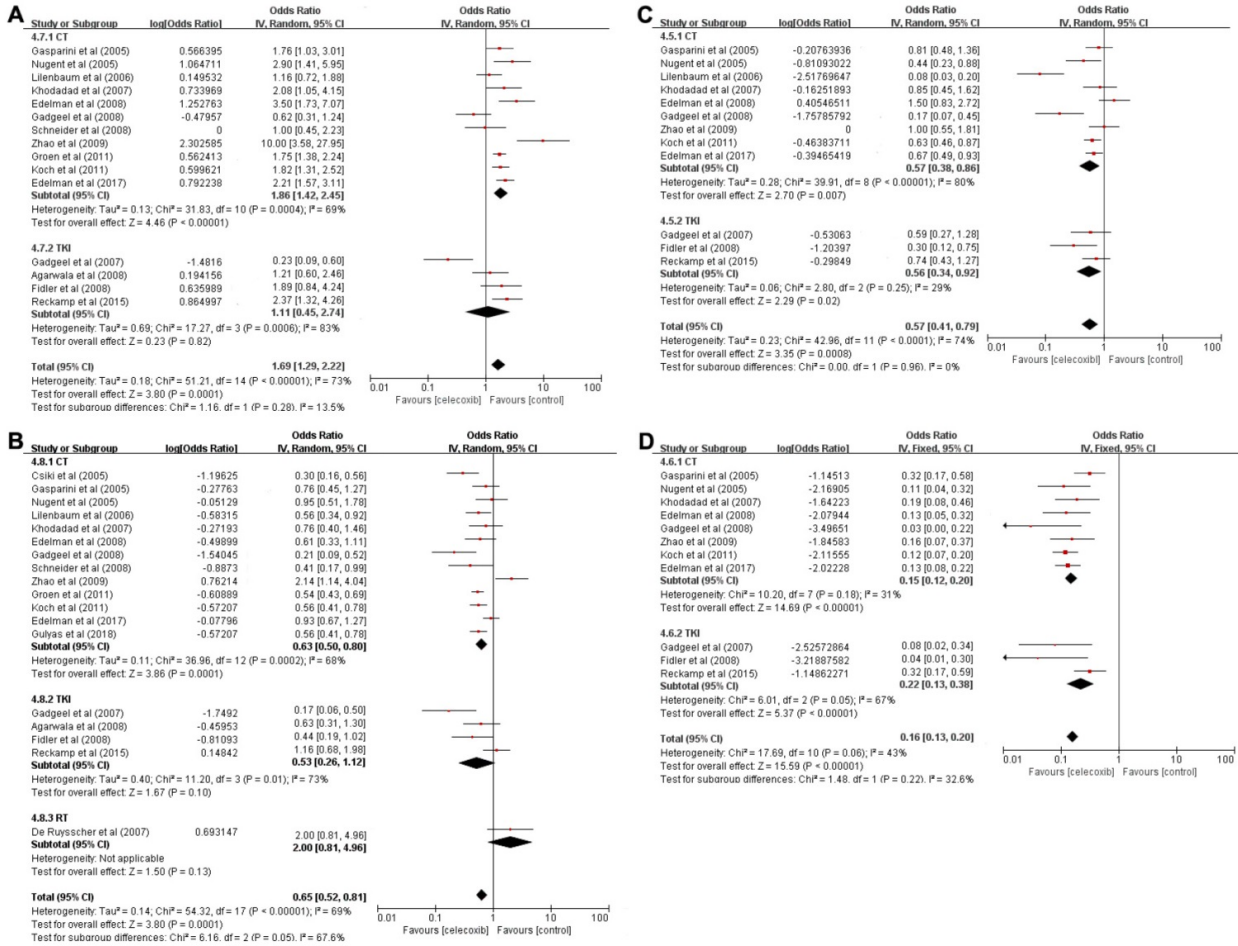
Forest plots of overall survival (OS) and progression-free survival (PFS), namely, OS-6 (A), OS-12 (B), PFS-6 (C), and PFS-12 (D), for celecoxib treatment combined with systematic therapy. Note: (A) OS-6; (B) OS-12; (C) PFS-6; (D) PFS-12. Abbreviations: IV: inverse variance; CI: confidence interval; CT: chemotherapy; TKIs: tyrosine kinase inhibitors; RT: radiotherapy.

A total of sixteen trials including 691 patients were used to analyze OS-6 (OR=0.62, 95% CI 0.55-0.69, P<0.001) (Table 2), as determined by the random-effects model (I^2^=73%, P<0.001) (Figure 3A). A pooled analysis of sixteen studies containing 454 patients was performed to assess OS-12 (OR=0.39, 95% CI 0.34-0.46, P<0.001) (Table 2), with the random-effects model (I^2^=70%, P<0.001) (Figure 3B).

Based on the significantly improved OS-6 and OS-12 in NSCLC patients, a subgroups analysis was carried out to explore whether the improved OS was associated with different treatment patterns. According to the subgroup analysis, chemotherapy in combination with celecoxib resulted in higher OS-6 (OR=0.65, 95% CI 0.59-0.71, P<0.001) than the celecoxib and TKI combination (OR=0.53, 95% CI 0.31-0.73, P=0.82). Moreover, the chemotherapy group was clearly different from the TKI group in term of OS-6 (65% vs 53%, P=0.0392). A similar result was found for OS-12; data pooled from sixteen studies that compared celecoxib with chemotherapy to celecoxib monotherapy showed a significant difference in OS-12 (OR=0.39, 95% CI 0.33-0.45, P<0.001). In contrast, the aggregated OS-12 of celecoxib in combination with TKIs or radiotherapy showed no significant improvement (P=0.1, P=0.13), with a highly significant difference between radiotherapy and chemotherapy or TKIs (67% vs 39%, P=0.0359 or 67% vs 35%, P=0.0321) and with no significant difference between chemotherapy and TKIs in terms of one-year survival (39% vs 35%, P=0.5751). These results revealed that celecoxib combined with chemotherapy was superior to celecoxib combined with TKIs or radiotherapy in terms of the 6-month OS and 1-year OS.

A total of 276 patients enrolled in 12 trials who received celecoxib and systemic treatment were analyzed to evaluate PFS-6 (OR=0.36, 95% CI 0.29-0.44, P<0.001) (Table 2), as determined with the random-effects model (I^2^=74%, P<0.001) (Figure 3C). Eleven trials with 87 patients assessed the OR for PFS-12 (OR=0.14, 95% CI 0.12-0.17, P<0.001) (Table 2) after celecoxib combined with systemic treatments, with no significant between-study heterogeneity (I^2^=43%, P<0.001) (Figure 3D).

The PFS analysis indicated that celecoxib significantly differed from systemic therapy alone; thus, we further conducted subgroup analyses of PFS-6 and PFS-12 to analyze whether the results were associated with a specific treatment pattern. The results suggested that the PFS-6 for celecoxib plus chemotherapy was significantly improved (OR=0.36, 95% CI 0.28-0.46, P=0.007). Equally, celecoxib in combination with TKIs significantly increased PFS-6 (OR=0.36, 95% CI 0.25-0.48, P=0.02), and no difference was found between the chemotherapy and TKI groups (36% vs 36%, P=1.000). Likewise, there was an increase in PFS-12 (OR=0.13, 95% CI 0.11-0.17, P<0.001) with celecoxib added to chemotherapy. Similarly, compared with TKIs alone, celecoxib plus TKIs showed markedly enhanced PFS-12 (OR=0.18, 95% CI 0.16-0.28, P<0.001). No significant difference was observed between the two groups in terms of PFS-12 (13% vs 18%, P=0.6038).

**Figure 4:**
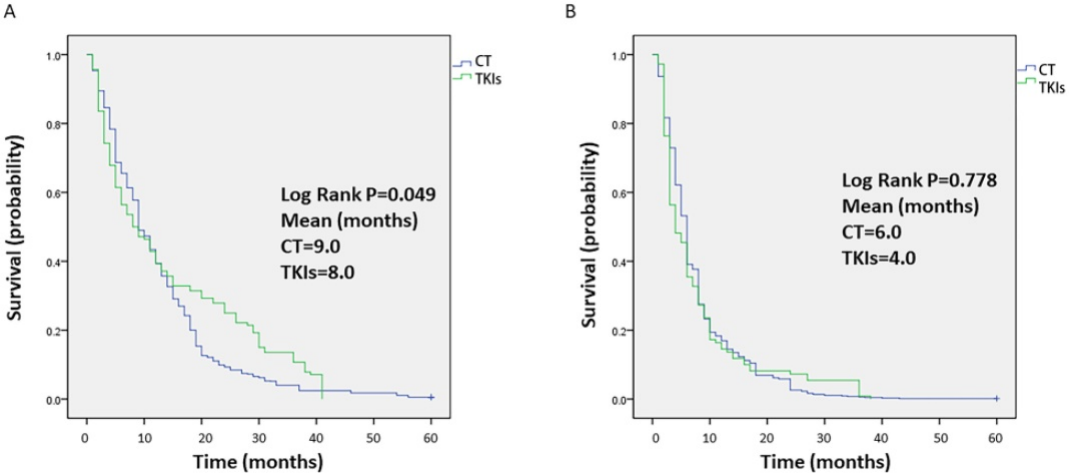
Kaplan-Meier analysis of the overall survival (OS) (A) and progression-free survival (PFS) (B) of patients treated with celecoxib in combination with chemotherapy or tyrosine kinase inhibitors. Note: (A) OS and (B) PFS.

Data on survival were collected from each study, and the median OS time was 9 months or 8 months for celecoxib in combination with chemotherapy or TKIs, respectively. The OS outcomes were significantly different between the two groups (P=0.049, Figure 4A). In terms of short OS (within 9 months), the results favored celecoxib combined with chemotherapy. However, for one-year OS, celecoxib combined with TKIs was slightly better than celecoxib combined with chemotherapy, but the survival rate was less than 40%. In addition, the median PFS time was 6 months or 4 months for celecoxib combined with chemotherapy or TKIs, respectively. No difference was apparent between the two groups regarding PFS (P=0.778, Figure 4B).

### Subgroup analyses

The efficacy of celecoxib combined with antitumor therapy among different groups was ascertained. According to the range of publication years, further subgroup analyses were performed to evaluate the clinical benefits for PFS, OS and ORR. The data are provided in Table S1.

We set the publication cut-off year to 2010, and divided the publications into two groups: between January 1, 2010 and December 31, 2019 (≥2010), and from January 1, 2001 and December 31, 2010 (<2010). Based on the results of the subgroup analysis, celecoxib significantly increased the ORR (OR=0.22, 95% CI 0.17-0.29, P<0.001), with a significant difference in the ORR between before the year of 2010 and after the year of 2010 (38% vs 62%, P<0.001). In addition, a pooled analysis of the PFS found a similar result for celecoxib combined with antitumor treatment on the PFS-6 (OR=0.36, 95% CI 0.29-0.44, P<0.001) and PFS-12 (OR=0.14, 95% CI 0.12-0.17, P<0.001) in advanced NSCLC patients before the year of 2010 and after the year of 2010, with no significant difference between the two groups (47% vs 53%, P=0.3370 or 45% vs 55%, P=0.3374).

However, a further subgroup analysis of OS showed a significant correlation with time, i.e., there was no significant decrease in OS-6 before the year of 2010 (OR=0.61, 95% CI 0.49-0.71, P=0.06), but the OS-6 was significantly increased with celecoxib after the year of 2010 (OR=0.66, 95% CI 0.62-0.69, P<0.001). A similar phenomenon was observed for the 12-month OS, where celecoxib combined with antitumor therapy improved the OS-12 ≥2010 (OR=0.38, 95% CI 0.30-0.47, P=0.007), while the OS-12 was not changed <2010 (OR=0.42, 95% CI 0.34-0.57, P=0.06). Further, a comparison of the rates before the year of 2010 and after the year of 2010, we found that there was a significant difference in the OS-6 and OS-12 between the two groups (P<0.001 and P=0.032, respectively).

### Toxicities

We also evaluated the side effects of celecoxib in combination with anticancer treatment in advanced NSCLC. Grade III or higher toxicities were significantly increased with celecoxib combined with systemic therapy. The common toxicities caused by celecoxib were assessed in a subgroup analysis to explore whether the side effects were caused by different treatment patterns. The results revealed that celecoxib combined with chemotherapy increased AEs, including anemia (OR=0.12, 95% CI 0.07-0.22), leukopenia (OR=0.27, 95% CI 0.16-0.42), thrombocytopenia (OR=0.22, 95% CI 0.12-0.40), neutropenia (OR=0.31, 95% CI 0.21-0.43), nausea/vomiting (OR=0.05, 95% CI 0.02-0.13), diarrhea (OR=0.04, 95% CI 0.02-0.05), fatigue/asthenia (OR=0.08, 95% CI 0.02-0.30), and cardiac ischemia (OR=0.02, 95% CI 0.01-0.05). Similarly, the celecoxib plus TKI combination treatment for advanced NSCLC improved toxicities (grade ≥ III): anemia (OR=0.03, 95% CI 0.02-0.06), leukopenia (OR=0.08, 95% CI 0.05-0.14), nausea/ vomiting (OR=0.13, 95% CI 0.01-0.68), diarrhea (OR= 0.17, 95% CI 0.01-0.64), fatigue/Asthenia (OR=0.10 95% CI 0.07-0.15), and cardiac ischemia (OR=0.03, 95% CI 0.00-0.20). These results indicated that the side effect profile of celecoxib combined with chemotherapy or TKIs was more serious than that of previous monotherapy. No significant differences were found in AEs between the two groups: anemia (P=0.8003), leukopenia (P=0.7319), nausea/vomiting (P=1.0000), diarrhea (P=0.6204), fatigue/asthenia (P=0.9278) and cardiac ischemia (P=0.9745). These results suggested that celecoxib combined with chemotherapy or TKIs increased hematological toxicity and cardiovascular events. The toxicity data are shown in Table 3.

### Publication bias

The Begg's funnel plot and Egger's test were applied to estimate the publication bias of the outcomes (Figure S1). The data for OS-6, OS-12, PFS-6, and PFS-12 did not show asymmetry. Egger's test established a linear regression equation by using the normalized effect scale as the dependent variable and the accuracy of the effect estimators as the independent variable. In our analysis, we used the standard error (SE) as the independent variable and the standardized estimate (logor) as the dependent variable. Similarly, OS-6, OS-12, PFS-6, and PFS-12, as evaluated with Egger's test, indicated no significant publication bias (P>0.05). However, publication bias was found in the ORR (P<0.001) (Table 4). However, further analysis with the trim-and-fill test demonstrated that the assessments were not affected.

## Discussion

In this study, we investigated the clinical efficacy of celecoxib in combination with anticancer therapy for patients with advanced NSCLC. We performed a systematic review and meta-analysis to clarify the optimal combination with different treatments. Our data showed that celecoxib combined with chemotherapy or TKIs significantly improved the ORR, with no statistically significant difference in the ORR between the two groups. In terms of OS, celecoxib combined with chemotherapy was superior to TKIs and radiotherapy. However, the efficacy of chemotherapy combined with celecoxib was not satisfactory because this combination treatment increased the occurrence of AEs. Additionally, celecoxib in combination with chemotherapy or TKIs significantly improved PFS, and the chemotherapy group had an advantage over the TKI group regarding PFS.

Studies in mice and cultured cells have shown that COX-2 has an important role in the induction and development of cancers, and COX-2 is upregulated in lung cancer and correlates with tumor angiogenesis and apoptosis.^38^,^39^ Furthermore, COX-2 overexpression can reduce host immunity and regulate cell adhesion to enhance tumor invasion and metastasis.^40^,^41^ Therefore, COX-2 is considered a target, and the reversal of its effects on tumor multidrug resistance via the COX signaling pathway is mediated by inhibiting COX-2 in cancer cells. Accordingly, COX-2 inhibitors combined with chemotherapy significantly inhibit the growth of human lung cancer cells.^42^,^43^ Celecoxib, a highly selective COX-2 inhibitor, can be used in combination with chemotherapy for patients with NSCLC after previous monotherapy failure.^38^ Our previous study.^32^ confirmed a PFS benefit after systematic treatment combined with celecoxib in patients with advanced NSCLC. However, the optimal combination with different treatments remains unclear. Therefore, further quantitative assessment was conducted to compare the efficacy of celecoxib combined with different anticancer therapies in patients with advanced NSCLC.

Our meta-analysis showed a significant increase in the ORR of celecoxib combined with chemotherapy or TKIs in patients with advanced NSCLC (P<0.001), yet the celecoxib combination with radiotherapy rarely improved the ORR (P=0.13), with no significant difference between the chemotherapy group and TKI group in terms of the ORR (P=0.4779). Indeed, COX-2 inhibitors combined with chemotherapy could enhance the antitumor activity of chemotherapy and inhibit the growth of lung cancer cells.^44^ Additionally, a phase I clinical trial has indicated that the celecoxib plus erlotinib combination treatment might improve tumor response in patients with NSCLC harboring EGFR gene mutations. The potential mechanism is that EGFR-dependent MAPK/ERK signaling pathways might be activated via prostaglandin E2 (PGE2) metabolized by COX-2, which further downregulates E-cadherin expression to promote the epithelial-mesenchymal transition (EMT).^45^,^46^ In summary, celecoxib combined with chemotherapy or TKIs can improve local control, but no improvement is evident when celecoxib is combined with radiotherapy.

In this meta-analysis, celecoxib in combination with chemotherapy improved OS-12 (OR=0.39, 95% CI 0.33-0.45, P<0.001), with a significant difference between chemotherapy and radiotherapy; however, OS-12 was not prolonged by celecoxib combined with radiotherapy or TKIs. No difference was found between the chemotherapy group and the TKI group. OS-6 was enhanced by chemotherapy combined with celecoxib (OR=0.65, 95% CI 0.59-0.71, P<0.001), while an OS-6 enhancement was not evident with celecoxib plus TKI combination treatment (OR=0.53, 95% CI 0.31-0.73, P=0.82). The difference was apparent between the chemotherapy group and the TKI group regarding OS-6 (P=0.0392). However, in terms of OS, the median OS time for patients treated with celecoxib plus chemotherapy was only 9 months, indicating that some patients benefited from this combination treatment, especially patients selected based on COX-2 expression in the tumor, because celecoxib inhibited the COX-2 and PGE2 levels induced by chemotherapy in tumors. Interestingly, preclinical studies have revealed that chemotherapy results in the upregulation of COX-2, which synthesizes high levels of PGE2.^47^,^48^ PGE2 promotes angiogenesis and enhances tumor metastasis. Similarly, Csiki et al. have shown that low-level urine PGE-M significantly prolongs survival compared to high-level urine PGE-M.^49^ In addition, clinical trials from Edelman and colleagues have demonstrated that patients with low COX-2 protein levels have better OS times than those with high COX-2 protein levels. Based on these observations, COX-2 inhibitors can prevent the growth of human cancer cells by reducing the levels of COX-2 and PGE2 in tumors and enhancing the activity of chemotherapy drugs.^50^ However, celecoxib in combination with TKIs did not evidently improve OS (including OS-6 and OS-12; this may be due to the EGFR mutational status in patients with advanced NSCLC, which was undefined in our study. Gadgeel et al. have provided strong evidence that combining celecoxib with EGFR-TKIs can improve the clinical efficacy of treatment for patients with EGFR mutations but not for patients with wild-type EGFR.^51^ In terms of cancers with EGFR mutations, a similar observation showed that patients with mutations in exon 19 of EGFR had a higher median OS rate than patients with mutations in exon 21 of EGFR in response to gefitinib and erlotinib.^6^ Another randomized controlled trial (RCT) reported similar results involving more mutant isomers (exon 19, exon 21, and T790M).^52^ Consequently, these observations suggest that the EGFR mutational status and mutation type have a significant impact on the reliability of the results. Although this analysis was limited by the quality of published studies and the number of patients, we ought to cite larger, well-designed RCTs of celecoxib combined with TKIs to refine the population of patients with EGFR mutations for more reliable results. Equally, a significant increase in OS-12 was not evident with the celecoxib and radiotherapy combination treatment. Some preclinical studies have supported the idea that COX-2 inhibitors in combination with radiotherapy provides potential benefits for patients with advanced NSCLC.^53^,^54^ However, there is no clear conclusion on the survival rate because we examined only one relevant study.^55^ A clearer conclusion must be made with additional high-quality articles related to radiotherapy combined with celecoxib.

Our study confirmed that celecoxib combined with chemotherapy or TKIs could prolong PFS-6 (P=0.007 or P=0.02) and PFS-12 (P<0.001), without significant differences between the two groups. Trials in xenograft tumor models have shown that selective COX-2 inhibitors can inhibit the growth of lung cancer cells and enhance the efficacy of chemotherapy for NSCLC.^26^ Additionally, our previous study, which has been published, reported that COX-2 inhibitors combined with chemotherapy might partially improve the PFS of patients with advanced NSCLC.^32^ Celecoxib in combination with TKIs prolongs PFS, which is consistent with the results of a phase II clinical trial evaluating erlotinib combined with celecoxib. This RCT showed that PFS was improved in patients with COX-2 overexpression.^30^ The potential mechanism was as follows: the EGFR signaling pathway is evidently correlated with COX-2 pathways, and PG produced by COX-2 may activate the EGFR signaling pathway and increase the levels of COX-2 and PGE2 in tumor cells. Therefore, the EGFR-TKI plus celecoxib combination treatment might be most efficient in patients with EGFR-mutant NSCLCs and function by decreasing the expression of COX-2 and PGE2.^51^,^56^-^58^ Although the improved outcome in terms of PFS was especially evident when celecoxib was combined with chemotherapy or TKIs, the median PFS of the chemotherapy group was superior to that of the TKI group. Regarding OS with the three anticancer therapies combined with celecoxib, the combination of celecoxib with chemotherapy was best, but the median OS time with this combination treatment was only 9 months. We may further explore the survival benefits of celecoxib combined with anticancer therapy, with more high-quality and carefully designed experimental studies.

The results suggested that celecoxib significantly increased the ORR. We conducted a subgroup analysis to explore whether the efficacy of celecoxib combined with antitumor therapy was related to the publication time. The results showed that the increase in the ORR was more obvious in publications after the year of 2010 (38% vs 62%, P<0.001), compared to before 2010. Compared to 10 years ago, the proportion of patients with advanced NSCLC who have been enrolled in studies has increased. These studies have shown that COX-2 has enhanced the antitumor activities of traditional chemotherapeutic drugs *in vivo* and *in vitro* when patients were treated with celecoxib.^59^ Regarding PFS (PFS-6 or PFS-12) with the three anticancer therapies combined with celecoxib, no significant difference was found between the two group mentioned above, i.e., studies published before or after 2010. However, in terms of OS, including OS-6 or OS-12, a significant difference was found between studies published before the year of 2010 and those after the year of 2010. In fact, although the data from 10 years ago may differ from the current data, due to the detailed criteria for the selection of the studies, the research scheme and the population were consistent across all studies. Because there were no unified standards for specific study designs during the time range, we set 2010 as the publication cut-off date, which may have thereby increased the choice bias and influenced the results.

Finally, we assessed the toxicities of celecoxib in combination with anticancer treatments for advanced NSCLC. Our data confirmed a significant increase in grade 3 and 4 toxicities. The subgroup analysis indicated that adding celecoxib to chemotherapy increased the occurrence of hematological toxicities and nonhematological toxicities, including leucopenia, thrombocytopenia, cardiovascular events, and diarrhea. Liu et al. suggested that COX-2 inhibitors inhibited angiogenesis after chemotherapy, which was correlated with the inhibition of VEGF and platelet-derived factors.^60^ This may be a potential cause of the increased incidence of leukopenia and thrombocytopenia.^61^ Similarly, celecoxib combined with TKIs increased anemia, leukopenia, cardiovascular toxicity and other side effects. However, only 1-2 relevant studies were included in our study, and the number of patients was so small that the P-value was significantly affected. Additionally, the results showed that celecoxib in combination with chemotherapy or TKIs increased the incidence of cardiovascular events, but only two studies described these events; these studies described seven patients, six of whom were treated with chemotherapy plus celecoxib and one patient who was treated with celecoxib combined with TKI. A similar study indicated that the long-term use of celecoxib increased the risk of cardiovascular disease in patients with advanced NSCLC.^62^ However, in contrast to the previous study, other studies did not find that the COX-2 inhibitors used in the treatment of NSCLC raised the risk of cardiovascular events.^63^,^64^ There have been controversies over the cardiovascular toxicity of celecoxib added to anticancer therapy, and a large number of doctors and scientists have carried out research on COX-2 inhibitors to actively explore these key questions. In summary, celecoxib combined with chemotherapy or TKIs increases the side effects of treatment for advanced NSCLC. The dose of celecoxib should be strictly controlled in clinical applications.

Our study had several limitations. First, this study had significant heterogeneity, and the clinical and methodological differences between studies might be responsible for the high heterogeneity. For example, the ratio class calculation method was conducted to pool the outcome data in the meta-analysis of a single rate, which obviously expanded the meaning of the P-value. Second, we only involved trials published in English, resulting in language bias. Third, a significant difference was found between the number of patients in RCTs and that in single-arm studies, which weakened the authenticity of the results. Additionally, the results were statistically significant, making them easier to publish, thereby leading to funnel asymmetry, such as publication bias, which was obviously found in terms of the ORR (P<0.001). Moreover, chemotherapy regimens were included as first-line or second-line treatment, and we did not perform subgroup analysis according to the treatment line, resulting in exaggerated effectiveness. We also did not conduct a stratification analysis of patients who were resistant to radiotherapy or chemotherapy. Furthermore, due to insufficient data, patients with advanced NSCLC with EGFR mutations were unstratified. Accordingly, we should select the specific target medicine on the basis of patients with EGFR-mutant NSCLC; otherwise, we will easily misestimate the effect. Although this meta-analysis is not perfect, it still has some guiding significance for clinical practice.

## Conclusion

Overall, celecoxib combined with chemotherapy or TKIs significantly improved the ORR, with no statistically significant difference in the ORR between the two groups. In terms of OS, celecoxib combined with chemotherapy was superior to the combination with TKIs or radiotherapy. Additionally, celecoxib in combination with chemotherapy or TKIs significantly improved PFS, and the chemotherapy group had an advantage over the TKI group regarding PFS. However, the combination of celecoxib and chemotherapy increased hematological toxicities and cardiovascular events. Therefore, additional high-quality studies are needed to confirm these conclusions.

## Supplementary Material

Supplementary figures and tables.

## Figures and Tables

**Table 1:** Characteristics of the patients in constituent trials

Study (year)	Phase	Country	Study period	Treatment line	Age (years)	ECOG PS	Sample size	Treatment Pattern	Treatment program	Dosage and length of celecoxib
									Drugs/Dosage(mg/m2)/days /Frequency of cycles	
Lilenbaum et al (2006)	II	America	2002 to 2003	Second	37-84	0-1	67	CT + Celecoxib	Irinotecan 100 + gemcitabine 1000/Irinotecan 60 + docetaxel 35/d 1, 8/3 weekly	400 mg, bid, to PD
De Ruysscher et al (2007)	II	Netherland	2003 to 2004	First	41-86	0-2	21	RT + Celecoxib	Radiotherapy 60 Gy, 2 Gy/d, 5 times /weekly	400 mg, bid, 2 years
Edelman et al (2008)	II	America	2003 to 2004	First	NR	0-2	45	CT + Celecoxib	Carboplatin AUC 5.5/d 1 + gemcitabine 1000/d 1, 8 + zileuton 600 mg/qid	400 mg, bid, to PD or 6 cycles
Groen et al (2011)	III	Netherland	2003 to 2007	First	33-84	0-2	281	CT + Celecoxib	Carboplatin AUC 6.0/d 1 + docetaxel 75 /d 1/3 weekly	400 mg, bid, to PD and ≤3 years
Koch et al (2011)	III	Sweden	2003 to 2006	First	37-85	0-2	158	CT + Celecoxib	Carboplatin/cisplatin + a third-generation drug/3 weekly	400 mg, bid, 1 year
Reckamp et al (2015)	II	America	2007 to 2011	Second	30-80	0-1	54	TKIs + Celecoxib	Erlotinib 150 mg/day	600 mg, bid, to PD
Edelman et al (2017)	III	America	2010 to 2013	First	36-89	0-2	154	CT + Celecoxib	Carboplatin AUC 6.0 + pemetrexed 500/d 1/Carboplatin AUC 5.5/d 1+ gemcitabine 1000 /d 1, 8/3 weekly	400 mg, bid, to PD
Csiki et al (2005)	II	America	2001 to 2003	Second	37-85	0-1	56	CT + Celecoxib	docetaxel (75 mg/m2) day 1; q3w	400 mg, bid, to PD
Gasparini et al (2005)	II	Italy	2002 to 2004	Second	30-77	0-2	58	CT + Celecoxib	Paclitaxel (80 mg/m2) weekly for 6 weeks; q8w	400 mg, bid, to PD
Nugent et al (2005)	II	US	2001 to 2003	Second	44-77	0-2	39	CT + Celecoxib	Docetaxel (75 mg/m2) day 1; q3w	400 mg, bid, to PD
Reckamp et al (2006)	I	America	2003 to 2005	Second	35-94	0-1	22	TKIs + Celecoxib	Erlotinib (150 mg/day)	200 to 800 mg, bid, to PD
Gadgeel et al (2007)	II	America	2003 to 2004	Second	35-74	0-2	27	TKIs + Celecoxib	Gefitinib (250 mg/day)	400 mg, bid, to PD
Khodadad et al (2007)	II	Tehran-Iran	2003 to 2005	First	28-70	0-2	37	CT + Celecoxib	Paclitaxel (200 mg/m2) + carboplatin (AUC 6) day 1; q3w	200 mg, bid, to PD
Agarwala et al (2008)	II	Indiana	2004 to 2004	First	19-93	0-1	31	TKIs + Celecoxib	Gefitinib (250 mg/day)	400 mg, bid, to PD
Fidler et al (2008)	II	America	NR	Second	46-81	0-2	26	TKIs + Celecoxib	Erlotinib (150 mg/day)	400 mg, bid, to PD
Gadgeel et al (2008)	II	America	2001 to 2004	First	51-82	0-2	34	CT + Celecoxib	Docetaxel (36 mg/m2) weekly	400 mg, bid, to PD
Schneider et al (2008)	II	America	2001 to 2002	Second	41-76	0-2	24	CT + Celecoxib	Docetaxel (75 mg/m2) day 1; q3w	400 mg, bid, to PD
Zhao et al (2009)	II	China	2005 to 2007	First	56	0-2	44	CT + Celecoxib	Cisplatin 80 mg/m2 day 1,2 + gemcitabine 1250 mg/m2 or novelbine 25 mg/m2 day 1, 8 or docetaxol 75 mg/m2 day 1; q3w	400 mg, bid, to PD

Abbreviations: AUC: area under the curve; CT: chemotherapy; ECOG PS: Eastern Cooperative Oncology Group performance status; NR: not reported; PD: progression disease; RT: radiotherapy; TKIs: tyrosine kinase inhibitors.

**Table 2:**
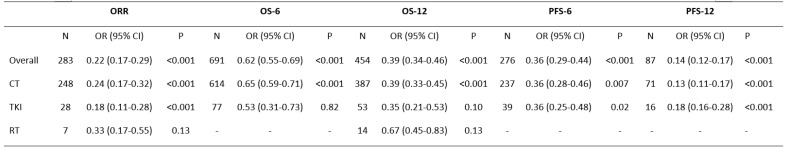
Meta-analysis of the clinical endpoints in advanced NSCLC for the treatment of celecoxib combined with systematic therapy

Abbreviations: OR: odds ratio; CI: confidence interval; ORR: overall response rate; OS-6: 6-month overall survival; OS-12: one-year overall survival; PFS-6: 6-month progression-free survival; PFS-12: 12-month progression-free survival; CT: chemotherapy; TKIs: tyrosine kinase inhibitors; RT: radiotherapy.

**Table 3:** Meta-analysis of the toxicities between patients treated with celecoxib in combination with chemotherapy or tyrosine kinase inhibitors. The test for heterogeneity is indicated by the I^2^ value.

Treatment pattern		CT		TKI	Heterogeneity	CT vs TKI
Toxicity	N	OR (95% CI)	N	OR (95% CI)	I2	P
Hemoglobin	7	0.12 (0.07-0.22)	1	0.03 (0.02-0.06)	91%	0.8003
Leucopenia	3	0.27 (0.16-0.42)	1	0.08 (0.05-0.14)	94%	0.7319
Neutropenia	9	0.31 (0.21-0.43)	NA	NA	89%	NA
Platelets	5	0.22 (0.12-0.40)	NA	NA	94%	NA
Nausea/ vomiting	5	0.05 (0.02-0.13)	2	0.13 (0.01-0.68)	89%	1.0000
Diarrhoea	3	0.04 (0.02-0.05)	4	0.17 (0.01-0.64)	94%	0.6204
Fatigue/ Asthenia	5	0.08 (0.02-0.30)	3	0.10 (0.07-0.15)	90%	0.9278
Cardiac ischaemia	1	0.02 (0.01-0.05)	1	0.03 (0.00-0.20)	0%	0.9745

Abbreviations: CI: confidence interval, CT: chemotherapy; TKIs: tyrosine kinase inhibitors; NA: not available; N: number of included studies; OR: odds ratio.

**Table 4:** Meta-analysis of publication bias of the outcomes for the treatment of celecoxib combined with systematic therapy.

Outcomes	P-value
**ORR**	<0.001
**OS-6**	0.699
**OS-12**	0.868
**PFS-6**	0.159
**PFS-12**	0.150

Abbreviations: ORR: overall response rate; OS-6: 6-month overall survival; OS-12: one-year overall survival; PFS-6: 6-month progression-free survival; PFS-12: 12-month progression-free survival.
