# Supplementary material for: Comparison of the benefits of celecoxib combined with anticancer therapy in advanced non-small cell lung cancer: A meta-analysis
Source: J Cancer. 2020 Jan 20;11(7):1816–27. doi: 10.7150/jca.35003 (PMC7052875; doi:10.7150/jca.35003)
Supplement: Supplementary file 1 — Supplementary figures and tables. [file jcav11p1816s1.pdf]

Figure S1 Funnel plot of the overall response rate (ORR), 6-month overall survival rate (OS-6), one-year overall survival rate (OS-12), 6-month progression-free survival rate (PFS-6), and 12-month progression-free survival rate (PFS-12) for the treatment of celecoxib combined with systematic therapy.

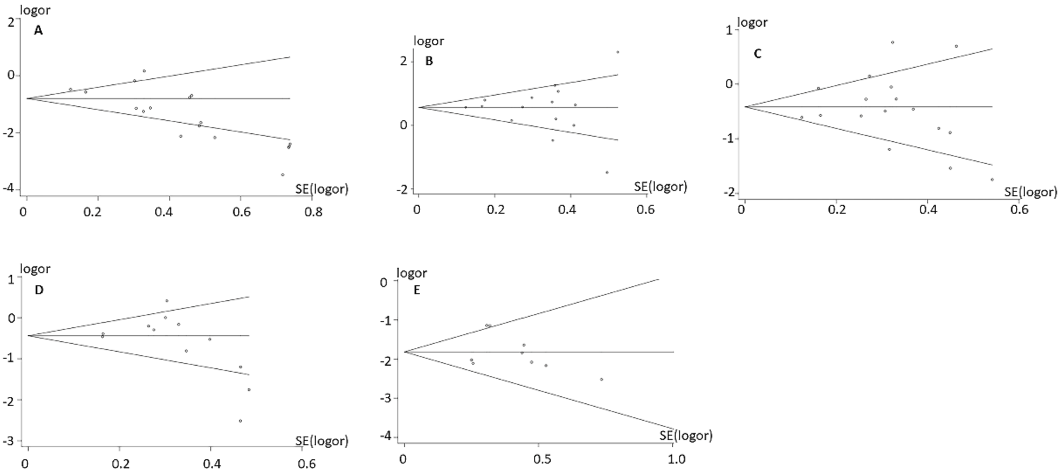

Note: (A) ORR; (B) OS-6; (C) OS-12; (D) PFS-6; (E) PFS-12.

Abbreviations: SE: standard error; OR: odds ratio.

Figure S2 Forest plots of the overall response rate (ORR) for celecoxib treatment combined with systematic therapy.

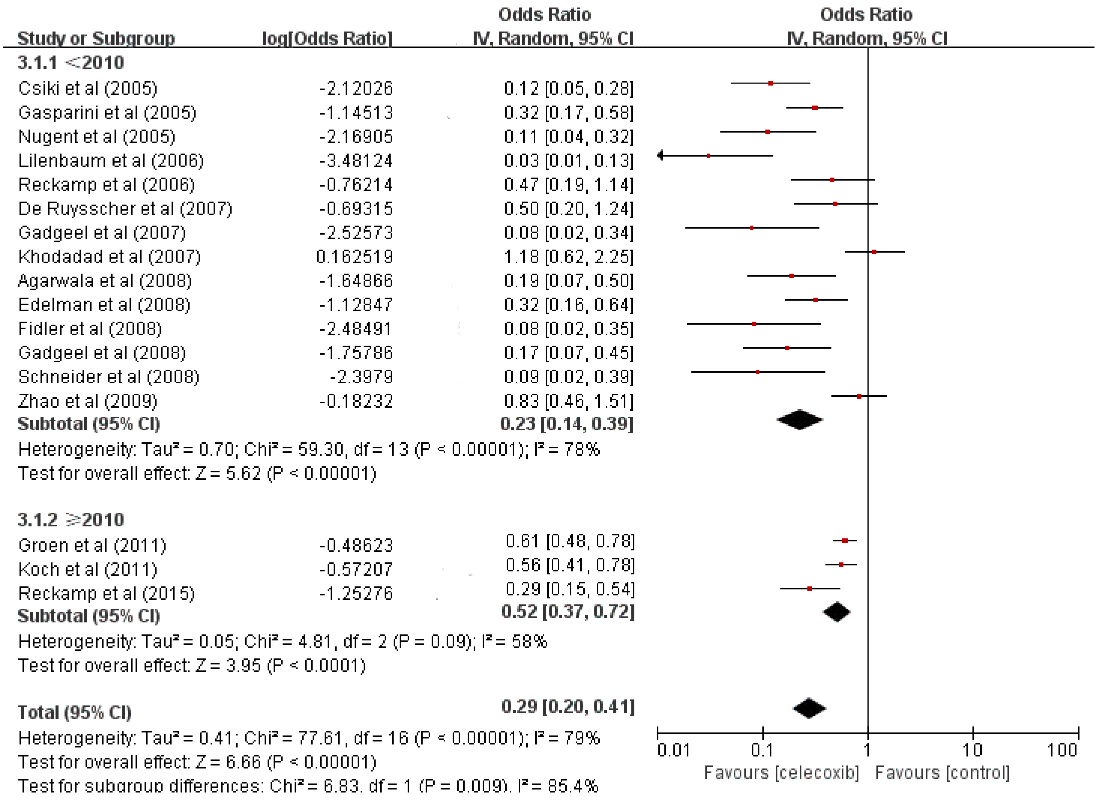

Note: (A) OS-6; (B) OS-12; (C) PFS-6; (D) PFS-12.

Abbreviation: OR: odds ratio; IV: inverse variance; CI: confidence interval; ≥2010: between January 1, 2010 and December 31, 2019; <2010: from January 1, 2001 and December 31, 2010.

Figure S3 Forest plots of overall survival (OS) and progression-free survival (PFS), namely, OS-6 (A), OS-12 (B), PFS-6 (C), PFS-12 (D), for celecoxib treatment combined with systematic therapy.

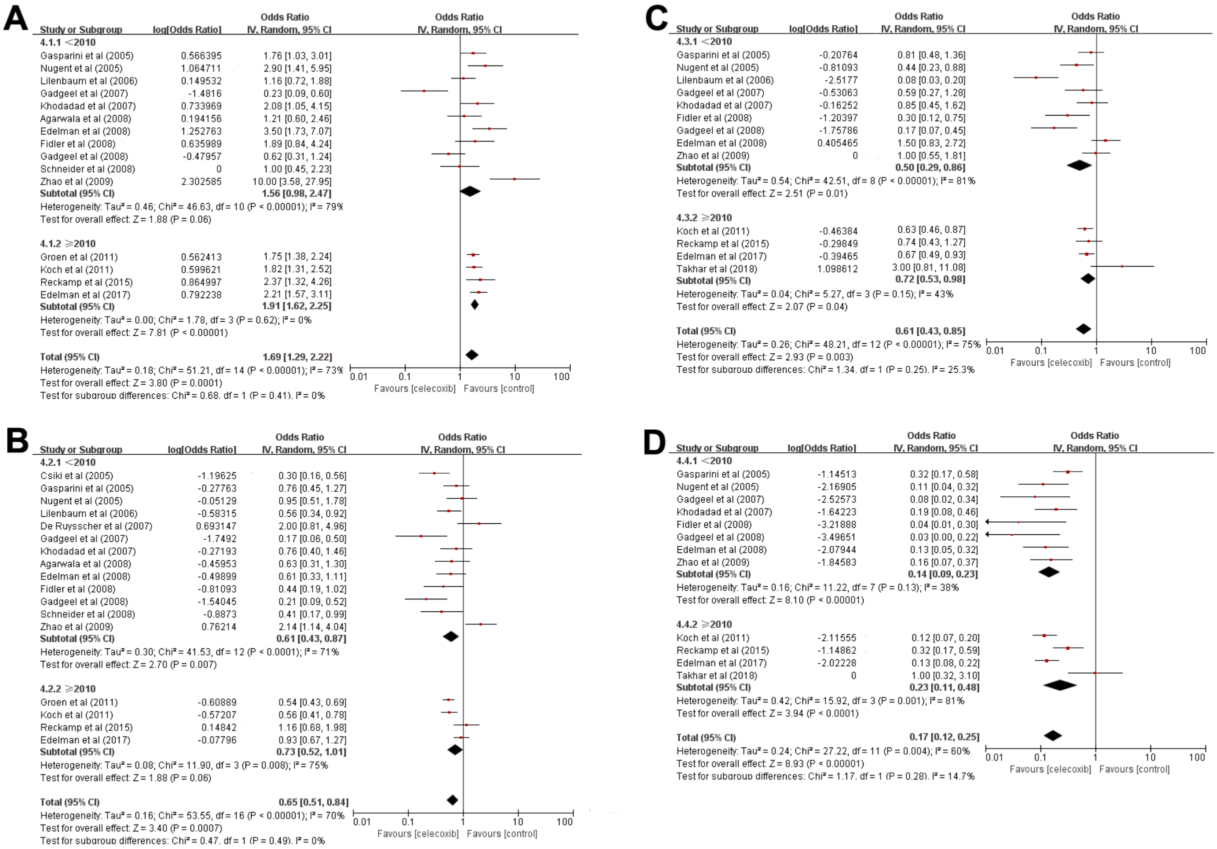

Note: (A) OS-6; (B) OS-12; (C) PFS-6; (D) PFS-12.

Abbreviations: IV: inverse variance; CI: confidence interval; ≥2010: between January 1, 2010 and December 31, 2019; <2010: from January 1, 2001 and December 31, 2010.

Table S1 Meta-analysis of the clinical endpoints in advanced NSCLC for celecoxib treatment combined with systematic therapy.

	ORR			OS-6			OS-12			PFS-6			PFS-12		
	N	OR (95% CI)	P	N	OR (95% CI)	P	N	OR (95% CI)	P	N	OR (95% CI)	P	N	OR (95% CI)	P
**Overall**	283	0.22 (0.17-0.29)	<0.001	691	0.62 (0.55-0.69)	<0.001	454	0.39 (0.34-0.46)	<0.001	276	0.36 (0.29-0.44)	<0.001	87	0.14 (0.12-0.17)	<0.001
⩾**2010**	107	0.19 (0.12-0.28)	<0.001	266	0.61 (0.49-0.71)	=0.06	195	0.38 (0.30-0.47)	=0.007	130	0.33 (0.22-0.46)	=0.01	39	0.15 (0.11-0.19)	<0.001
<**2010**	176	0.34 (0.27-0.42)	<0.001	425	0.66 (0.62-0.69)	<0.001	259	0.42 (0.34-0.50)	=0.06	146	0.40 (0.35-0.45)	<0.001	48	0.14 (0.11-0.)	<0.001

Abbreviations: OR: odds ratio; CI: confidence interval; ORR: overall response rate;OS-6: 6-month overall survival; OS-12: one-year overall survival;PFS-6: 6-month progression-free survival; PFS-12: 12-month progression-free survival; ⩾2010: between January 1, 2010 and December 31, 2019; <2010: from January 1, 2001 and December 31, 2010.
